# Model-Based Therapeutic Correction of Hypothalamic-Pituitary-Adrenal Axis Dysfunction

**DOI:** 10.1371/journal.pcbi.1000273

**Published:** 2009-01-23

**Authors:** Amos Ben-Zvi, Suzanne D. Vernon, Gordon Broderick

**Affiliations:** 1 Department of Chemical and Materials Engineering, University of Alberta, Edmonton, Alberta, Canada; 2 The CFIDS Association of America, Charlotte, North Carolina, United States of America; 3 Department of Medicine, University of Alberta, Edmonton, Alberta, Canada; Northwestern University, United States of America

## Abstract

The hypothalamic-pituitary-adrenal (HPA) axis is a major system maintaining body homeostasis by regulating the neuroendocrine and sympathetic nervous systems as well modulating immune function. Recent work has shown that the complex dynamics of this system accommodate several stable steady states, one of which corresponds to the hypocortisol state observed in patients with chronic fatigue syndrome (CFS). At present these dynamics are not formally considered in the development of treatment strategies. Here we use model-based predictive control (MPC) methodology to estimate robust treatment courses for displacing the HPA axis from an abnormal hypocortisol steady state back to a healthy cortisol level. This approach was applied to a recent model of HPA axis dynamics incorporating glucocorticoid receptor kinetics. A candidate treatment that displays robust properties in the face of significant biological variability and measurement uncertainty requires that cortisol be further suppressed for a short period until adrenocorticotropic hormone levels exceed 30% of baseline. Treatment may then be discontinued, and the HPA axis will naturally progress to a stable attractor defined by normal hormone levels. Suppression of biologically available cortisol may be achieved through the use of binding proteins such as CBG and certain metabolizing enzymes, thus offering possible avenues for deployment in a clinical setting. Treatment strategies can therefore be designed that maximally exploit system dynamics to provide a robust response to treatment and ensure a positive outcome over a wide range of conditions. Perhaps most importantly, a treatment course involving further reduction in cortisol, even transient, is quite counterintuitive and challenges the conventional strategy of supplementing cortisol levels, an approach based on steady-state reasoning.

## Author Summary

The hypothalamic-pituitary-adrenal (HPA) axis is one of the body's major control systems helping to regulate functions ranging from digestion to immune response to metabolism. Dysregulation of the HPA axis is associated with a number of neuroimmune disorders including chronic fatigue syndrome (CFS), depression, Gulf War illness (GWI), and posttraumatic stress disorder (PTSD). Objective diagnosis and targeted treatments of these disorders have proven challenging because they present no obvious lesion. However, the body's various components do not work in isolation, and it is important to consider exactly how their interactions might be altered by disease. Using a relatively simple mathematical description of the HPA axis, we show how the complex dynamical behavior of this system will readily accommodate multiple stable resting states, some of which may correspond to chronic loss of function. We propose that a well-directed push given at the right moment may encourage the axis to reset under its own volition. We use model-based predictive control theory to compute such a push. The result is counterintuitive and challenges the conventional time-invariant approach to disease and therapy. Indeed we demonstrate that in some cases it might be possible to exploit the natural dynamics of these physiological systems to stimulate recovery.

## Introduction

The hypothalamic-pituitary-adrenal (HPA) axis constitutes one of the major peripheral outflow systems of the brain, serving to maintain body homeostasis by adapting the organism to changes in the external and internal environments. It does this by regulating the neuroendocrine and sympathetic nervous systems as well modulating immune function [1]. Through regulation of these systems, the HPA axis initiates and coordinates responses to physical stressors; such as infection, hemorrhage, dehydration, thermal exposure and to neurogenic stressors; such as fear, anticipation and fight or flight.

Many aspects of the organization and function of the HPA axis have been characterized in clinical and laboratory studies revealing a number of component feedback and feed forward signaling processes. Stress activates the release of corticotropin-releasing hormone (CRH) from the paraventricular nucleus (PVN) of the hypothalamus. The release of CRH into the hypophysial-portal circulation in turn acts in conjunction with arginine vasopressin on CRH-R1 receptors of the anterior pituitary stimulating the rapid release of adrenocorticotropic hormone (ACTH). ACTH then is released into the peripheral circulation and stimulates the release of the glucocorticoid cortisol from the adrenal cortex by acting on the receptor MC2-R (type 2 melanocortin receptor). Cortisol enters the cell and binds to the glucocorticoid receptor present in the cytoplasm of every nucleated cell; hence the widespread effects of glucocorticoids on practically every system of the body including endocrine, nervous, cardiovascular and immune systems.

To keep HPA axis activity in check, glucocorticoids also exert negative feedback at the hypothalamus and pituitary glands to inhibit the synthesis and secretion of CRH and ACTH, respectively. Moreover, glucocorticoid negative feedback causes a reduction in corticotroph receptor expression leading to a desensitization of the pituitary to the stimulatory effects of CRH on ACTH release. This negative feedback is also felt in the hippocampus where it exerts a negative influence on the PVN. A detailed review of the physiology and biochemistry of the HPA axis as well as it's know interactions with the immune system may be found in work by Silverman et al. [2].

A number of chronic diseases have been characterized by abnormalities in HPA axis regulation. These include major depression and its subtypes, anxiety disorders such as post-traumatic stress disorder, panic disorder and cognitive disorders such as Alzheimer's disease and minimal cognitive impairment of aging [3]. Dysregulation of the HPA axis has also been linked to the pathophysiology of Gulf War illness [4], post-infective fatigue [5], and chronic fatigue syndrome (CFS) [6],[7]. It is not clear what causes this dysregulation, but it is manifested in many HPA axis disorders as a hypercortisol or hypocortisol state. The existence of these separate and stable states is not surprising when one considers the multiple feedforward and feedback mechanisms that regulate the HPA axis. Systems such as this often display complex dynamics that readily accommodate multiple stable steady states which are known as attractors because the system is naturally drawn back to these resting states after perturbation. However, if the perturbation is of sufficient strength and duration, the system can be pushed away from a given resting state and into the basin of new attractor.

Though much is known about its components, one of the main difficulties in studying the behavior of the HPA axis has been in integrating the expansive body of published experimental information. Numerical models provide an ideal framework for such integration. Simple models of the HPA axis have been constructed using deterministic coupled ordinary differential equations [8],[9]. Though successful in reproducing some of the basic features of HPA axis dynamics these early models neglected to include feedback and feed-forward immune effector molecules and associated mechanisms. Linear approximations of some components lead to unrealistic predictions beyond a very narrow region of concentrations. In addition transport processes involved in the distribution of these chemical signals from the brain throughout the body were not modeled explicitly. This level of abstraction made direct comparison of simulation results to actual HPA axis chemistry and physiology highly tenuous. In a move towards increased fidelity Gupta et al. [10] introduced a more detailed description of glucocorticoid receptor dynamics enabling the latter to demonstrate bistability in HPA axis dynamics. As mentioned previously this theoretical proof of the existence of a second stable steady state is highly compatible with clinical observations. Moreover the abnormally low cortisol levels characterizing this stable resting state or basin of attraction are consistent with documented observations of hypocorticolism in patients with CFS [11], Gulf War illness and other similar conditions [12]–[14].

In this work we adopt the model proposed by Gupta et al. [10] as a recent and detailed representation of the HPA axis. On the basis of this model we propose a framework for estimating robust corrective measures for displacing the HPA axis from a chronic hypocortisol state back to a healthy state. Using model-based predictive control (MPC) methodology we demonstrate that it is possible to compute such treatment time courses while dealing with the inherently high level of uncertainty characteristic of biological systems. While this uncertainty might lead to compromises in efficiency, interventions can be computed that predict a positive outcome. Our analysis indicates that one such treatment could involve a pharmacologically induced reduction in cortisol forcing a build-up of ACTH. Upon reaching a specific threshold concentration of ACTH, the intervention is discontinued and the HPA axis will return to a healthy steady state under its own volition as this is now the closest attractor for the system.

## Methods

### The HPA Axis Model

A model of the HPA axis which includes glucocorticoid receptor and the dynamics of glucocorticoid receptor-cortisol interactions have been proposed by Gupta et al. [10]. This model is described by the following differential equations as System *H* (Eq. 1).
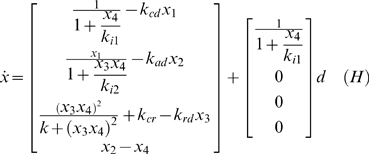
(1)


The system states are given as x = [x_1_; x_2_; x_3_; x_4_]^T^ and are described in Table 1. Note that the states in this model are scaled values, as described by Gupta et al. [10]. The system parameters are given by the vector p = [k_i1_; k_cd_; k_ad_; k_i2_; k_cr_; k_rd_; k]^T^. Nominal values for the system parameters are listed in Table 2. The variable d in System H is the stress term which describes the effect of stress (both physical and psychological) on the hypothalamus. This variable is seen as a disturbance that perturbs the System *H* from a steady-state value.

**Table 1:** Steady-state values for concentrations of CRH, ACTH, free GR and circulating cortisol.

State	Description	Stable Rest Points
x_1_	CRH concentration	(0.6261, 0.6610)
x_2_	ACTH concentration	(0.0597, 0.0513)
x_3_	Free GR concentration	(0.0809, 0.5629)
x_4_	Cortisol concentration	(0.0597, 0.0513)

**Table 2:** Parameter settings for the differential equation model of the HPA axis proposed by Gupta et al. [10].

Parameter	Description	Value
k_i1_	Inhibition constant for CRH synthesis	0.100
k_cd_	CRH degradation constant	1.000
k_i2_	Inhibition constant for ACTH synthesis	0.100
k_ad_	ACTH degradation constant	10.000
k_cr_	GR synthesis constant	0.050
k_rd_	GR degradation constant	0.900
k	Inhibition constant for GR synthesis	0.001

### Control of the HPA Axis System

In this first analysis the HPA axis system is considered under idealized conditions where all parameters are assumed constant and precisely known. In addition, the states x are assumed known as a function of time with no measurement error and the control action is implemented perfectly. The approach taken for choosing an optimal control is based on the Model Predictive Control (MPC) framework [15]–[17]. Under this framework, an objective function of the manipulated and measured variables is defined. Typically the objective function is a mathematical expression which corresponds to engineering objectives or underlying system constraints. The input computed under the MPC framework is the one in a class of permissible inputs that minimizes the chosen objective function.

In this work it is assumed that the variable to be manipulated for treatment is the rate of addition or removal of cortisol from circulation. To model this control action, System *H* is augmented with a control term u in the equation for cortisol (x_4_) (Eq. 2). Note that System *Hu* is affine with respect to the control action and the disturbance.
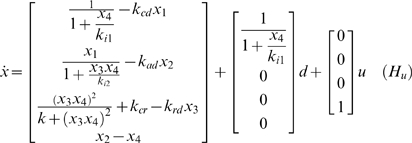
(2)


To avoid dangerous destabilization of the HPA axis by the application of control action *u(t)* we define the following penalty function to enforce minimal departure from normal ACTH (x_2_) and cortisol (x_4_) levels even though we purposely manipulate circulating cortisol to perturb the system *H_u_*.
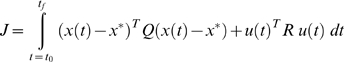



Where t_0_ and t_f_ are the start and end time of the optimization horizon, λ is a tuning parameter taking values from zero to one and x_2_^*^ and x_4_^*^ are the healthy steady-state concentrations of ACTH and cortisol, respectively. *R* is a penalty assigned to the input and *Q* is the penalty assigned to the state variables. R was chosen as 0 because the cost for therapy was considered negligible compared to the cost of ongoing disability. *Q* was chosen as follows because x_2_ and x_4_ are the only measured states.
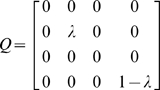



The resulting cost function can be written as:
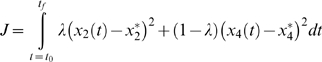
(3)


The parameter λ is used to penalize excessive imbalance of the other hormones (x_1_, x_2_, x_3_) in response to the control action applied to cortisol (x_4_). In this case, the objective of the controller is to bring the cortisol concentration to set point while minimizing the impact of the treatment on the other three states of the HPA axis. Any change in CRH (x_1_) or the glucocorticoid receptor (GR; x_3_) will be reflected in the concentration of ACTH (x_2_) by virtue of the coupled dynamics described by System H_u_. The tuning parameter λ can be selected to match the intensity of the desired treatment. A λ value of near zero will lead to a more intense treatment while a value of λ near one will lead to very conservative treatment. For proof of concept, a more direct treatment was favored in this work and a λ value of 0.01 was used throughout. Note that x_2_^*^ and x_4_^*^ correspond to the stable steady state of the unperturbed system (i.e., when *u* = 0). As a result, once the system has been brought to the healthy steady state it will stay at this steady state even if the external control action (treatment) is removed.

Typically, a treatment or control action is applied at discrete intervals. As a result, the objective function in Equation 3 was optimized with respect to a piece-wise constant input signal *x_4_(u(t))*. That is, the optimization procedure searched for an optimal input in the set U_c_ of all piecewise constant functions on 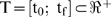 defined such that the input level may be changed every 1/2 scaled time unit. The optimization problem was therefore posed in Equation 4 as:
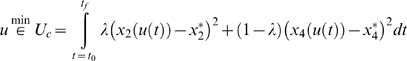
(4)


The initial condition, x(t = t_0_) is the steady state of the unperturbed system with d_0_ = 0. The optimal control, 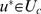 was computed using Matlab's built-in “fminsearch” function.

## Results

### Steady-State Analysis

The steady-state solutions for HPA axis model described above as System *H* can be computed by setting 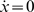 and treating the right side of System *H* as a set of four algebraic equations in the four unknowns {x_1_; x_2_; x_3_; x_4_}. Under this framework, the disturbance variable, d, is assumed to take on a constant value 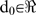. At steady state the system is therefore described by the following equations (Eq. 5–8).
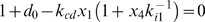
(5)

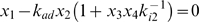
(6)

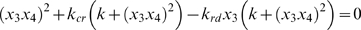
(7)

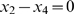
(8)


The above is a set of polynomials in x, with real coefficients, and maximum total degree of five. Equations 5 to 8 can be simplified using the theory of polynomial ideals [18]. Specifically, the latter can be reduced to the following set of equations (Eq. 9–12).
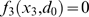
(9)

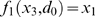
(10)

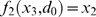
(11)

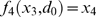
(12)


**Figure 1:**
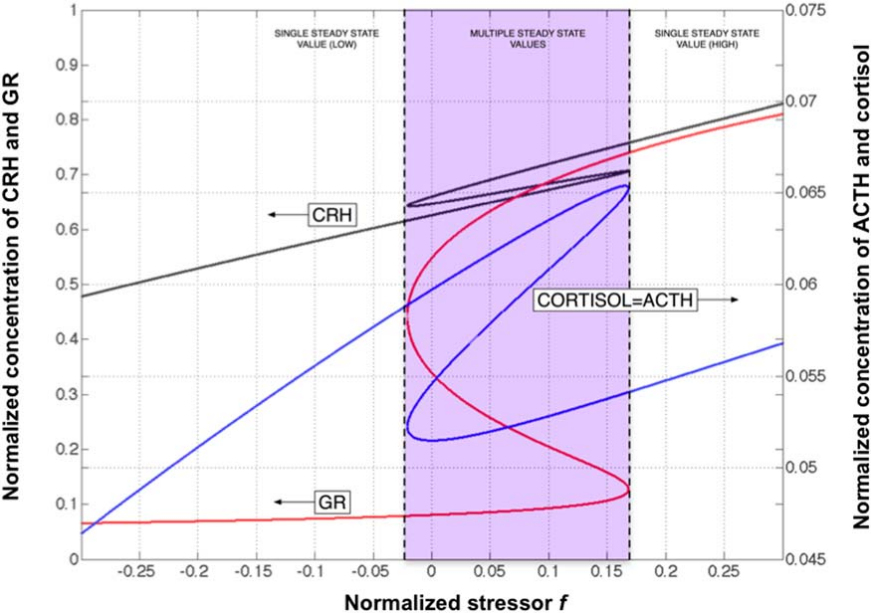
Steady states of the HPA axis system. Steady-state concentration of CRH (x_1_), ACTH (x_2_), GR (x_3_) and cortisol (x4) as a function of the external stressor *f* for the model expressed as system *H*. The system naturally accommodates 3 stable steady states at rest *f* = 0 and over a broad range of increasing values for *f*.

Therein f_3_ is a polynomial in x_3_ of degree seven, and f_1_, f_2_ and f_4_ are functions only of x_3_ and d_0_. The functions f_1_ to f_4_ can be computed using a symbolic algebra package such as Maple. For the nominal parameter values proposed in Gupta et al. [10] there are at most three real-valued solutions for x_3_ and these correspond to the roots of f_3_. Each root is a steady-state value for x_3_ and can be used to generate the corresponding values of x_1_, x_2_ and x_4_ given Equations 10 to 12. Note that at steady state x_2_ = x_4_ (Eq. 8). A plot of the steady-state values of x_1_, x_2_ and x_3_ as a function of d_0_ is shown in Figure 1.

**Figure 2:**
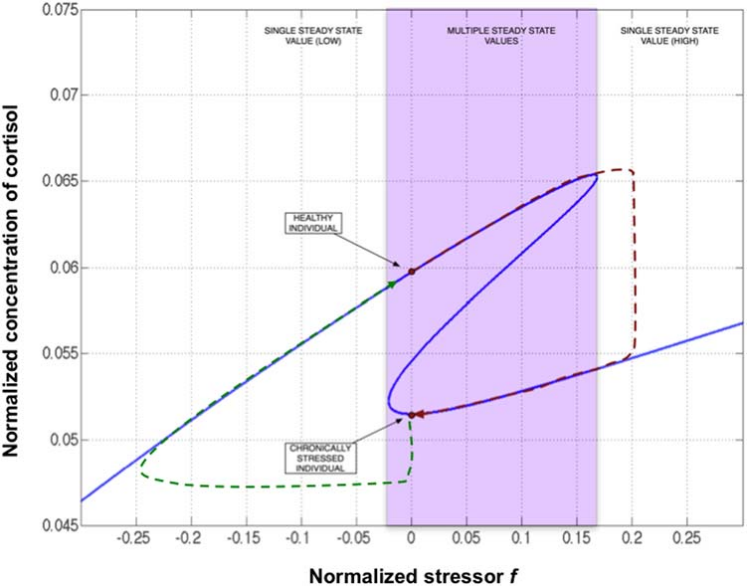
Migration of cortisol concentration from one stable point to another. Concentration of circulating cortisol plotted as a function of the external stressor *f*. A first idealized trajectory (red - -) describes the displacement of the system from rest to a peak cortisol concentration followd by an eventual lapse into a chronic hypocortisolic state. A second idealized trajectory (green - -) illustrates the effects of treatment. Here removal of cortisol can be thought of as a negative stress *f*. An increase in ACTH concentration of ∼30% above baseline serves as a signal that the treatment may be discontinued.

In this model of HPA axis dynamics a chronically stressed individual would occupy the stable steady state associated with a depressed cortisol concentration (∼0.05) at rest or at d_0_ = 0. If a healthy person were subjected to extreme stress (i.e., d_0_>0.168) for an extended period of time their body would reach the only steady state available locally that is one corresponding to chronic stress. In other words, for values of d_0_ greater than 0.168, Equation (9) dictates that there is only one steady-state solution for free GR (x_3_) concentration as opposed to the 3 solutions available for 0≤d_0_<0.168. By virtue of Equation (12) this results in only one steady-state solution being available for cortisol (x_4_) for d_0_>0.168. When the stress is removed (i.e., d_0_ = 0), the body will stay at this new depressed steady-state value of cortisol concentration. This process is shown graphically in Figure 2 by the red dashed trajectory. According to this model the inability of the body to return to the healthy steady state is due to the fact that once the body establishes a new equilibrium it inherently seeks to stay near this point. In order to force the body to return to its original equilibrium its state must first be shifted to a point where the only stable condition in proximity is one corresponding to this original healthy state. Once this is done, the internal regulatory mechanisms of the body will ensure that this healthy stable point is achieved and maintained. This approach is illustrated in Figure 2 by the green dashed trajectory. The design of such a shift is presented in the following section.

### Redirecting the HPA Axis Using an Idealized Disturbance

As one might expect the assumptions of ideal control do not correspond to a physically realizable system. However, the analysis of the system under idealized conditions allows the study of possible treatments. Any practical treatment would then be a suboptimal solution as compared to the treatment under idealized conditions. This allows proposed treatments to be benchmarked and compared. In addition, the solution obtained under idealized conditions can serve as a qualitative guideline for the creation of a practical, although suboptimal, treatment. In engineering terms the objective of treatment is to succeed in bring the subject to the healthy steady-state target while exerting the smallest disturbance possible to the HPA axis. For example, even though we intend to manipulate circulating cortisol concentration it should not be allowed to decrease excessively because of the important role cortisol plays in regulating a number of cellular and physiological functions. To avoid such excess perturbations the concentration of ACTH has been included in the objective function of Equation 3.This concentration is more readily measured than that of either CRH or GR making ACTH a good candidate for monitoring the progress of a treatment.

**Figure 3:**
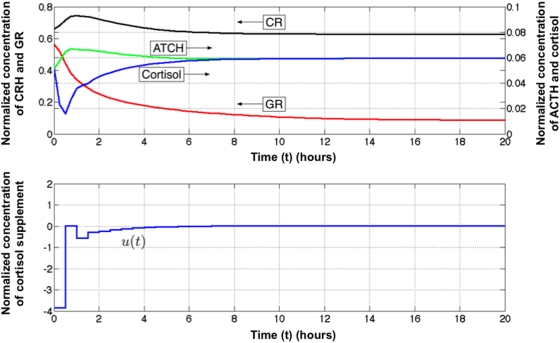
Idealized corrective control action. Concentrations of CRH (x_1_), ACTH (x_2_), GR (x_3_) and cortisol (x4) as a function of time in response to an ideal externally applied perturbation in cortisol *u(t)*. The negative supplement in cortisol signifies a pharmaceutical removal or inactivation of circulating cortisol. ACTH concentration serves to monitor the progress of the treatment which is discontinued when ACTH increases by ∼30% over baseline.

The optimal control solution that minimizes disruption of HPA axis function (Eq. 3) is shown in Figure 3 along with the system's overall trajectory. Note that the optimal input does indeed bring the system to the healthy steady-state point. This is done while maintaining a circulating cortisol concentration that is near the steady-state value with the exception of a rapid drop at the start of treatment. The optimal control solution as computed under the MPC framework has several key features. The cortisol concentration is rapidly dropped at the outset. Once this drop in cortisol concentration is achieved, the system requires little additional control action to come to steady state. This qualitative information can be used to formulate a suboptimal control strategy that will bring the system to the healthy steady state.

### A More Clinically Realistic Manipulation of the HPA Axis System

In this section a suboptimal control strategy is proposed for the HPA axis system. The goal of this strategy is to mimic the qualitative results of the MPC solution while being realizable in a clinical setting. The MPC solution suggests that manipulating cortisol concentration is a plausible strategy for redirecting the HPA axis to a healthy steady state. The key difficulty in applying this approach is determining when the cortisol concentration has been sufficiently lowered with regard to the other state variables to allow the system to return to a healthy equilibrium. That is, one must identify an observable event (corresponding to a measurable variable) which signals that the steady state of the system has shifted. In a clinical setting only ACTH and cortisol concentrations, corresponding to x_2_ and x_4_, respectively, can be readily measured. The availability of cortisol analogues makes it possible to manipulate x_4_ directly. Therefore as postulated previously (Eq. 3–4) ACTH (x_2_) can be used to determine when a change in available steady state or attractor has occurred. Under the MPC framework, most of the control action is expended near the initial time. In Figure 3 the external control action prescribed by MPC under ideal conditions and the response of ACTH (x_2_) are both plotted as a function of time. The value of x_2_ increases by about 30% as the system moves from the cusp of multiple candidate steady states to the basin of a single steady state. The following treatment is therefore proposed:

Treatment 1 The cortisol concentration in the system should be slowly decreased until ACTH levels (x_2_) have increased by more than 30% relative to the initial condition. Once this signal is observed, the system's own natural feedback control action should restore cortisol levels to normal.

**Figure 4:**
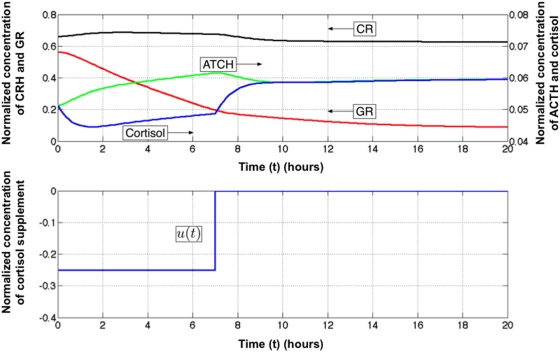
A suboptimal but clinically realistic control treatment. Concentrations of CRH (x_1_), ACTH (x_2_), GR (x_3_) and cortisol (x4) as a function of time in response to a suboptimal but more realistic externally applied perturbation in cortisol *u(t)*. Once again the negative supplement in cortisol signifies a pharmaceutical removal or inactivation of circulating cortisol. Note a less severe reduction in cortisol is applied over a longer period. The corresponding ACTH response is slower but the threshold concentration for cessation of treatment remains the same.

**Figure 5:**
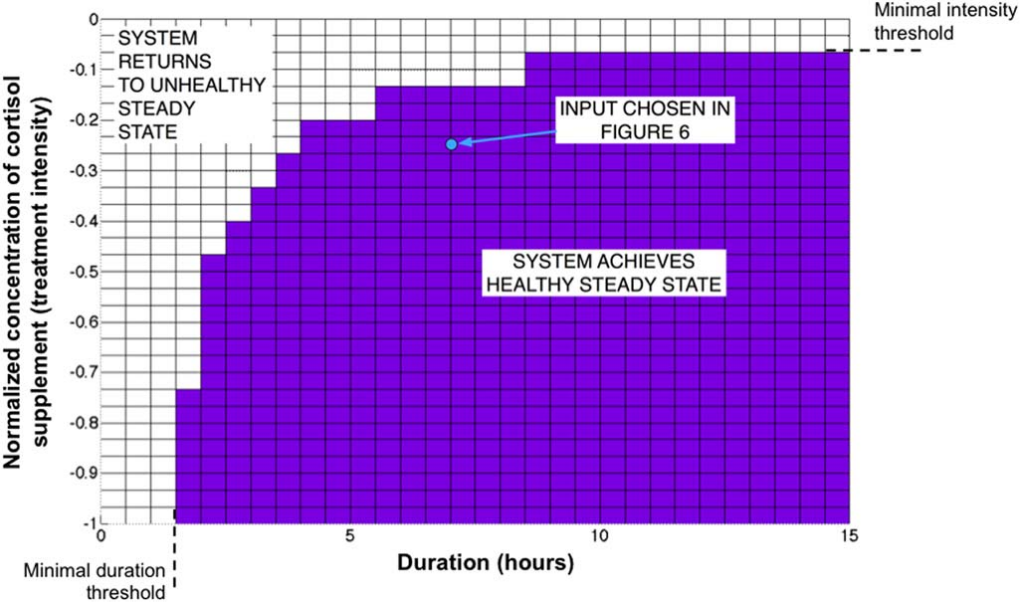
Balancing intensity and duration of treatment. Diagram of the minimal perturbation in normalized circulating cortisol *u(t)* as a function of duration of treatment. In one extreme instance the perturbation in cortisol would be so small that no treatment would be effective regardless of how much treatment prolonged. Conversely an excessively short treatment would also be ineffective regardless of the intensity of cortisol reduction.

Simulation results for Treatment 1 are shown in Figure 4. As indicated the system is brought to the healthy steady state via the suboptimal but more realistic treatment course. Furthermore, the drop in cortisol concentration is neither as severe nor as sharp as under naïve idealized MPC control. A positive outcome may also be obtained by applying even less severe levels of cortisol suppression and extending the duration of the treatment. Data presented in Figure 5 show that a combinations of treatment duration and cortisol suppression may be varied successfully over a large range. Nonetheless there exists a minimum level of cortisol suppression below which the treatment fails regardless of how long conditions are maintained. Conversely there also exists a minimal treatment duration below which even severe levels of cortisol suppression will prove unsuccessful in restoring normal hormone levels.

### Robustness Analyses

The results for Treatment 1 shown in Figure 4 are computed under nominal conditions. For the proposed treatment to be clinically useful, it must be effective over a wide variety of conditions, and parameter values. The robustness of the proposed approach to changes in the parameter values, initial conditions, and ambient stress level (i.e., value of d_0_) is examined in this section. A direct computational evaluation of robustness of Treatment 1 is difficult to implement. There are four initial conditions (x_1_(0); x_2_(0); x_3_(0); x_4_(0)), seven parameters, and one disturbance variable (d_0_). A simulation study where each variable (initial condition, parameter and disturbance) is evaluated at a nominal, high and low values, would require, at a minimum 3^12^ = 531,441 simulations. Even if these simulations were completed, the choice of high, low and nominal value for each variable would be difficult to justify using available data. An alternative approach analyzing robustness analysis is to study the asymptotic behavior of System *H_u_*. Let the concentration of cortisol (x_4_) be manipulated so that the product of cortisiol and GR concentrations (x_3_x_4_) is constant. Under these conditions, the asymptotic value of glucocorticoid receptor concentration GR (x_3_) is obtained from Eq. 7 as:
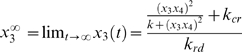
(13)


The asymptotic value or GR concentration x_3_∞ has a minimum as a function of cortisol concentration (x_4_) at x_4_ = 0. That is, if one were to lower the cortisol concentration to zero one would obtain the lowest possible steady-state value for GR and this value would be:
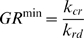
(14)


At the steady-state point given by x_4_∞ = 0 and x_3_∞ = k_cr_/k_rd_ the unique asymptotic solution for CRH (x_1_) and ACTH (x_2_) is given by
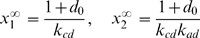
(15)


It should be noted that the values for GR, CRH and ACTH identified in Equations 14–15 represent an asymptotic minimum for the externally controlled system (System *H_u_*). For the closed loop HPA axis system it represents the minimum achievable cortisol concentration. Note that this equilibrium point is only achievable under external input. This result is independent of the trajectory of the input *u(t)* and is a property of the HPA axis. Moreover the solution in Equation 15 is unique indicating that only a single steady state exists at the minimum for x_4_→0 and that this state corresponds to a stable set of non-zero real-valued concentrations of CRH, ACTH and GR. This result confirms that reducing the cortisol concentration to a small enough positive value can indeed take the system to a single stable condition. This is regardless of the value of d_0_, parameters, or initial conditions. This condition will correspond to a healthy equilibrium value when treatment is administered in the absence of elevated levels of external stress d_0_. At high levels of the stressor d_0_ the success of the treatment would be short lived as we would simply be immediately re-administering the same insult originally responsible for the illness state. This is true regardless of whether the idealized or the suboptimal treatment approach is used.

## Discussion

Patients with CFS have been found to exhibit decreased adrenal response to ACTH stimulation and lower daily cortisol levels in plasma, urine and saliva [11],[19]. This is a chronic state in these patients and a detailed model by Gupta et al. [10] suggests that this condition may correspond to a stable steady state resulting from the higher order dynamics of the HPA axis. A robust treatment strategy was estimated using model-based predictive control methodology involving a controlled reduction of circulating cortisol concentration. This externally induced reduction in cortisol concentration is to be maintained until ACTH concentrations increase above a critical threshold. Though this treatment was derived through the use of a numerical model, it nonetheless provides an interesting conceptual strategy for treatment.

Cortisol output of the HPA axis can in reality be manipulated either directly or indirectly through several interventions. The most direct approaches involve (1) inhibition of cortisol synthesis at the level of the adrenal gland or (2) inhibition of CRH induced ACTH synthesis by the pituitary. Inhibitors of cortisol synthesis include pharmaceutical agents such as ketoconazole that have been used in limited human trials [20]. These are generally used in the treatment of hypercortisolism in patients and have been known to cause side effects including decreased androgen and aldosterone synthesis, elevated pregnenolone, nausea, fever, vomiting and occasionally hypoadrenalism and liver toxicity [21]. Likewise CRH antagonists have demonstrated antidepressant and anxiolytic properties in animal models of depression [22]. However only one phase II study involving the treatment of depressed patients with the CRH antagonist R121919 [23] has been completed thus far. The inhibition of CRH would not be useful in the current context as the proposed treatment aims to artificially stimulate an increase in ACTH concentration.

Indirect approaches to cortisol suppression focus on modulation of the biochemical feedback returning to the higher HPA axis from the immune system and the adrenal gland. Inflammatory events exert a positive immune system feedback to the HPA axis that is conducted via a number of pro-inflammatory cytokines for which several components of the HPA axis have receptors. Supported by immune, epidemiological and small-scale gene expression data [24], antagonists of the pro-inflammatory cytokine TNF-α have been used effectively in pilot clinical trials [25] to inhibit this positive feedback mechanism. The release of pro-inflammatory cytokines by the immune system can also be manipulated by altering the immune system's perception of circulating cortisol. Dexamethasone is a cortisol analogue that binds to GRII glucocorticoid receptor with a significantly higher affinity than that of endogenous cortisol [26],[27]. This saturation of the long-term receptor GRII with dexamethasone promotes down regulation of cortisol output by dampening the pro-inflammatory feedback signal. One possible explanation of hypocortisolism is an enhanced sensitivity to the negative feedback action of cortisol on the glucocorticoid receptors in what is termed dexamethasone hyper-suppression [12],[28]. Consistent with this mechanism, patients with CFS have shown a pronounced and prolonged suppression of salivary cortisol even after relatively low doses of dexamethasone [29]. Dexamethasone suppression has become a standard test procedure even though it has a significantly higher affinity for the GRII receptor over the GRI receptor, does not bind to corticosteroid binding globulin (CBG) and has a much longer half-life than endogenous cortisol. Recently Jerjes et al. [30] developed a similar protocol using prednisolone, a compound with physiological effects more similar to those of cortisol. Using 5 mg prednisolone, they achieved a 50% reduction in salivary cortisol in healthy subjects [30]. Similarly a 52% reduction in salivary cortisol and an 82% reduction in urinary cortisol were observed in CFS patients [31]. These relative levels of cortisol suppression are consistent with those required by this simulated treatment course and confirm that the system is indeed capable of accommodating such changes without ill effect.

Unfortunately as in strategies involving the direct inhibition of CRH, a reduction of positive feedback to the hypothalamus also leads to a reduction in ACTH synthesis by the pituitary. Recall that the proposed treatment requires the inhibition of the negative cortisol feedback without the removal of positive stimulation of ACTH production. This could be achieved by temporarily reducing the bioavailability of cortisol itself. Binding proteins and metabolizing enzymes have been identified for cortisol. Corticosteroid-binding globulin (CBG) regulates the concentration of free or active cortisol [32]. Oral oestrogen preparations have been shown to increase CBG levels [33]. In addition to CBG, the enzyme 11-β-hydroxysteroid dehydrogenase rapidly inactivates endogenous glucocorticoid hormones upon entry into the cell [34]. Similarly the multi-drug resistance (MDR) P-glycoprotein (Pgp) has been shown to control access of cortisol and corticosterone to the brain [35]. In all cases a reduction in the bioavailability of cortisol would limit the effect of negative feedback on ACTH synthesis without hampering the positive feedback from pro-inflammatory cytokines. ACTH would conceivably accumulate as a natural consequence of such an imbalance. ACTH could also be administered directly [36] under these conditions of reduced cortisol inhibitory feedback to accelerate the treatment course. Finally the treatment might also be administered at a time of day that corresponds to the natural circadian reduction in cortisol secretion.

It should be noted that although the model of HPA axis dynamics used in this work is currently the most credible model, it remains in many ways incomplete. For example, there is mounting data including observations of moderate hypocortisolism in depressed patients undergoing IFN-α therapy suggesting that GR receptor function not only affects the release of cytokines but is itself affected by these same cytokines [37]. The effects of cytokines and their signaling pathways on hormone signaling in general, and GR signaling in particular, is an important area of investigation regarding both the pathophysiology and treatment of inflammatory and neuropsychiatric diseases. To support these important aspects of HPA-immune signaling additional detail must be incorporated into the basic HPA axis model in particular at the level of the glucocorticoid receptors. Animal studies have not been exploited in this work but could undoubtedly serve as a basis for the construction of much more detailed models incorporating elements that are not readily measured in human experiments [38]. This is especially true of measurements at the hypothalamus. By the same token animal studies could be conducted to assess the tolerance of the overall system to more aggressive treatment and to determine a practical value for the parameter λ as well as the time period for monitoring and intervention. Certainly chronic and acute stressors such as the tail suspension test, the forced swim test and others have been used to produce depression-like symptoms in mice and have served to study hyperactivity of hypothalamic CRH neurons [39]. This model has also been used to test the effects of various anti-depressant therapies [40]. It should be noted however that CFS is characterized by a hypoactive rather than a hyperactive HPA axis [41]. Hyperactivity of hypothalamic CRH neurons observed in major depression produces a blunted ACTH response to further CRH challenge, likely reflecting a resultant down-regulation of pituitary CRH receptors [26],[37]. In contrast, subjects with CFS produce less cortisol in response to ACTH challenge but exhibit exaggerated ACTH responses to CRH [42]. This suggests that CFS hypocortisolism may arise from adrenal gland adaptation to a sensitized response at the level of the pituitary and/or the hypothalamus. While convincing murine models exist for the former condition [39],[40], we are not aware of an equivalent model that mimics the HPA axis hypoactivity observed in CFS. Models exist nonetheless that reproduce some facets of chronic fatigue. The most promising of these involve post-infectious fatigue induced in mice [43],[44]. No doubt as our understanding of the precise molecular signature of CFS improves so will the fidelity of our animal models enabling us to study CFS pathophysiology and treatment in earnest.

It is important to note however that while the specific treatment solution identified using MPC is model-dependent the general MPC framework is not. Therefore as more detailed models become available these can easily be exploited to improve a treatment course. Putting aside issues of model fidelity and completeness, the proposed MPC framework could still be exploited in a two-step treatment approach. In a first step data obtained from a standard dexamethasone test could serve to calibrate a simple lumped-parameter model capturing the overall HPA dynamics for a given subject. The calibrated model could then be used within the proposed MPC framework to estimate the most appropriate combination of dosage and duration of treatment for that same patient. Ultimately even if a given model is not entirely correct our robustness analysis shows that the desired outcome may be obtained reliably over a wide range of parameter values. This will be true as long as the structure of the model is valid.

### Conclusion

In conclusion we have demonstrated in this work the use of model-based predictive control methodology in the estimation of robust treatment courses for displacing the HPA axis from an abnormal hypocortisol steady state back to a normal function. Using this approach on a numerical model of the HPA axis proposed by Gupta et al. [10] a candidate treatment that displays robust properties in the face of significant biological variability and measurement uncertainty requires that cortisol be suppressed for a short period until ACTH levels exceed 30% of baseline. At this point the treatment may be discontinued and the HPA axis will progress to a stable attractor defined by normal hormone profiles. The concentration of biologically available cortisol could in principle be altered by binding proteins or metabolizing enzymes to inhibit negative feedback to the HPA axis without affecting the synthesis and accumulation of ACTH. Our analysis shows that this treatment strategy is robust and that a positive outcome can be obtained reliably for a wide range of treatment efficiencies.
